# Optimal patient selection beyond the current selection criteria for liver transplantation for unresectable colorectal liver metastasis

**DOI:** 10.1007/s00595-026-03297-x

**Published:** 2026-04-24

**Authors:** Masayuki Okuno, Takashi Ito, Ken Fukumitsu, Hiroto Nishino, Shinya Okumura, Katsunori Sakamoto, Satoshi Ogiso, Yoichiro Uchida, Takamichi Ishii, Etsuro Hatano

**Affiliations:** 1 Department of Surgery, Graduate School of Medicine, Kyoto University, 54 Shogoin-Kawahara-cho, Sakyo-ku, Kyoto, 606-8507 Japan; 2 Department of Surgery, Kyoto Katsura Hospital, Kyoto, Japan

**Keywords:** metastatic colorectal cancer, liver transplantation, recurrence

## Abstract

Liver transplantation (LT) is a potentially curative treatment option for patients with unresectable colorectal liver metastases (CRLM). Despite numerous reports of favorable overall survival following LT for CRLM by several high-volume transplant centers, recurrence-free survival remains far from satisfactory. Given the invasiveness of the procedure, the scarcity of deceased-donor organs, and the potential risks to living donors, optimal patient selection is crucial. Currently, several prognostic factors are used for selecting good candidates for LT for CRLM, including tumor burden, extrahepatic metastases, gene mutation profile, and response to chemotherapy. To this end, several scoring systems based on traditional prognostic factors have been proposed. This review also discusses the prognostic factors that are not incorporated into standard LT program criteria but may serve as novel eligibility markers to improve patient selection, including metabolic tumor volume assessed by PET/CT, ctDNA, and the presence of perihepatic lymph node metastases. Routine pathological assessment of perihepatic lymph nodes prior to LT may be especially warranted because a high liver tumor burden is correlated with an increased incidence of perihepatic lymph node metastases. Further clinical studies and results from ongoing LT trials are needed to validate the clinical utility of these proposed prognostic factors.

## Introduction

Approximately 18% of patients with colorectal cancer present with, or eventually develop liver metastases [1]. The standard of care for resectable colorectal liver metastases (CRLM) is surgical resection, with acceptable 5-year survival rates of 50–60%, which have been improving over recent decades [2, 3]. However, only 25% of patients with CRLM are candidates for curative hepatic resection because of extrahepatic disease spread or insufficient remnant liver volume following curative hepatectomy, even with multidisciplinary approaches such as portal vein embolization, two-stage hepatectomy or associating liver partition and portal vein embolization for staged hepatectomy [4]. Despite the reported efficacy of conversion surgery for initially unresectable CRLM after systemic chemotherapy with molecular targeting agents, up to 70% of patients who are treated with chemotherapy with the intention of conversion therapy still have unresectable disease [5, 6].

Liver transplantation (LT) is a potentially curative treatment option for highly selected patients with unresectable liver-only colorectal metastases involving both lobes and/or major vasculature. The Oslo group reported the largest experience to date of favorable LT outcomes for CRLM. Initially they conducted the prospective pilot SECA-I study and reported a 5-year overall survival (OS) rate of 60% following LT [7]. Subsequently, the prospective SECA-II trial demonstrated an increased 5-year OS rate of 80% [8, 9]. This survival improvement is likely attributable to the accumulation of knowledge about the prognostic factors for LT in CRLM, leading to more refined and appropriate patient selection. Several other transplantation programs for CRLM have been reported from North America, the United Kingdom, and Germany. An open-label, multi-institutional, single-arm clinical trial was launched in 2023 in Japan to evaluate the efficacy and safety of living-donor LT for patients with unresectable CRLM [10]. Recently, a randomized control TransMet trial showed significantly improved OS, with a 5-year OS of 73% for the LT plus chemotherapy group compared with 9% for the chemotherapy alone group within the per-protocol population [11]. In this context, LT is a promising treatment strategy for carefully selected patients with unresectable CRLM in several European and North American countries and has the potential to serve as an alternative to conventional treatment strategies for highly advanced CRLM in Japan.

Although a 5-year OS greater than 70% has been reported, the recurrence-free survival (RFS) rate after LT is low, with a 3-year RFS rate of approximately 35% [8, 11]. Moreover, the OS of patients with intrahepatic recurrence after LT for unresectable CRLM is only 14 months [12]. Considering the risks to living donors, the scarcity of transplant organs from deceased donors, and the invasiveness of the procedure for recipients, optimal patient selection is crucial. This review discusses the strategies for selecting optimal candidates for LT to treat CRLM.

### Scoring system

Several scoring systems for assessing individual patient prognoses after surgery for resectable CRLM have been reported (Table 1) [13–17]. The poor prognostic factors included in these scoring systems are the gene mutation profile, timing of metastases (synchronous or metachronous), disease-free interval, size and number of tumors, location of the primary tumor, primary tumor lymph node metastatic status, extrahepatic metastases at hepatectomy, CEA level, CA19-9 level, inflammatory response to the tumor, margin positivity, and progressive disease at liver resection. Compared with that after partial hepatectomy, the prognosis after LT for patients with CRLM is characterized by longer post-recurrence survival, and RFS is not an appropriate outcome parameter for assessing the efficacy of LT in patients with CRLM [18]. Other than Fong’s clinical risk score, these scoring systems for patients who undergo partial hepatectomy have not been evaluated in relation to whether they can be adopted for LT candidates. Therefore, a specific scoring system for LT, the Oslo score, was established based on the cohort in SECA-I study. This system is composed of the following pre-transplant factors: largest tumor diameter > 5.5 cm, plasma CEA level > 80 µg/L, time from surgery of the primary tumor to LT of < 2 years, and progressive disease on chemotherapy at the time of LT, and calculated by giving 1 point for each factor [7]. The Oslo group subsequently evaluated the performance of the Oslo score using the combined data of SECA-I and SECA-II and showed that the 5-year OS of patients with an Oslo Score of 0 to 2 was 67% compared with 17% for patients with an Oslo Score of 3 to 4. The median disease-free survival (DFS) of patients with an Oslo Score of 0 to 2 was longer at 19 months than at 3 months for patients with an Oslo Score of 3 to 4, and all patients with an Oslo Score of 3 to 4 had a relapse within the observational period. Moreover, they reported the applicability of Fong’s clinical risk score to patients who underwent LT for CRLM [19]. Fong’s clinical risk score (0 to 5 points) is calculated by giving 1 point for each of the following pretransplant characteristics: synchronous metastatic disease (< 12 months from diagnosis), a lymph node–positive primary, > 1 lesion, size > 5 cm, and CEA > 200 µg/L [13]. Although this scoring system was established using data from patients with CRLM who underwent partial hepatectomy, it demonstrated effective risk stratification for DFS, OS, and post-recurrence survival, even for patents with CRLM who underwent LT. As an Oslo score of 0 or a Fong’s clinical risk score of 1 is associated with a 10-year survival of more than 80% [19], and patients meeting these criteria represent highly selected candidates who may derive the greatest benefit from LT for unresectable CRLM. It should be noted that current LT programs for CRLM, including those of the Oslo group and TransMet trial, do not employ the Oslo score as formal inclusion criterion, but rather use it as an indicator of tumor burden. Therefore, further clinical studies are required to establish the utility of such scoring systems for strict patient selection for LT in clinical practice.

**Table 1:** Scoring systems for predicting survival after surgery for patients with colorectal liver metastases

Author	Year	Procedure	Prognostic factors	Predicting outcome
Fong (12)	1999	Hepatectomy	Node-positive primary tumor	OS
			Disease-free interval <12 months	
			Multiple hepatic tumors	
			Largest hepatic tumor >5 cm	
			CEA level >200 ng/ml	
Malik (13)	2007	Hepatectomy	Number of metastases ≥8	DFS and OS
			Presence of IRT	
Beppu (14)	2012	Hepatectomy	Synchronous metastases	DFS
			Primary lymph node positive	
			Number of tumors (solitary, 2-4, ≥5)	
			Largest tumor diameter >5 cm	
			Presence of extrahepatic metastases	
			CA19-9 level >100 U/mL	
Brudvik (16)	2019	Hepatectomy	Node-positive primary tumor	OS
			Size of largest liver metastasis >50 mm	
			RAS mutant	
Dasari (15)	2023	Hepatectomy	Age ≥65 years	OS
			ASA-PS class III/IV	
			Site of primary tumor (Transverse, right, rectum, left)	
			Primary lymph node status (N2, N1/N0)	
			Synchronous metastases	
			Number of liver tumors >5	
			Two stage hepatectomy	
			Resection margin of liver (R2, R1, R0)	
			No adjuvant chemotherapy	
			Major complication	
			Response to chemotherapy (PD, SD/PR/CR)	
Hagness (18)	2013	LT	Largest liver lesion >5.5 cm	OS
			CEA level >80 ng/ml	
			Time from surgery of primary tumor to LT <2 years	
			Progressive disease on chemotherapy	

CEA, carcinoembryonic antigen; IRT, inflammatory response to tumor; CA19-9, carbohydrate antigen 19-9; ASA-PS, American Society of Anesthesiologists-performance status; PD, progressive disease; SD, stable disease; PR, partial response; CR, complete response; LT, liver transplantation; OS, overall survival; DFS, disease-free survival

IRT is defined as an elevated C-reactive protein concentration (>10 mg/L) or a neutrophil/lymphocyte ratio of >5:1

### Biological factors

Primary tumor sidedness in colorectal cancer has emerged as an important prognostic factor. Several previous studies have reported that patients with left-sided stage III colorectal cancer have better RFS and OS after curative resection than those with right-sided disease [20, 21]. Similarly, among patients with unresectable stage IV colorectal cancer treated with systemic chemotherapy, right-sided primary tumors have been consistently associated with shorter OS than left-sided tumors [22, 23]. For patients with resectable CRLM, primary tumor sidedness has also been shown to influence survival after partial hepatectomy. McCracken et al. reported significantly longer OS following hepatectomy for patients with CRLM originating from left-sided primary tumors than right-sided primary tumors [24]. Similarly, Gasser et al. demonstrated superior OS after hepatectomy for patients with CRLM from left-sided versus right-sided primary tumors [25]. Collectively, these findings indicate that primary tumor sidedness is a relevant prognostic factor for stage III and IV colorectal cancer. With respect to patients with unresectable CRLM, who are candidates for LT, the Oslo group reported an association between primary tumor sidedness and post-transplant outcomes. Specifically, patients with left-sided primary tumors had significantly longer PFS and OS after LT than those with right-sided primary tumors [18]. Moreover, among patients with a high tumor burden, defined as ≥ 9 liver metastases or a maximum tumor diameter ≥ 5.5 cm, a trend toward a higher 5-year OS rate was observed among patients undergoing LT than among those treated with portal vein embolization followed by hepatectomy (72.4% vs. 53.1%, *p* = 0.08) [26]. To date, no other LT studies, including the TransMet trial, have evaluated the prognostic impact of primary tumor sidedness on survival after LT. Given that primary tumor sidedness is closely associated with distinct molecular profiles, such as higher frequencies of PIK3CA, PTEN, BRAF, and KRAS mutations in right-sided tumors, and more frequent APC and TP53 mutations, as well as EGFR and HER2 amplification in left-sided tumors, the extent to which sidedness itself independently influences post-transplant outcomes remains controversial [27]. Further studies are required to identify whether primary tumor sidedness should be incorporated as an eligibility criterion for LT in patients with unresectable CRLM.

The interval between primary tumor resection and LT, or between the diagnosis of CRLM and LT, is also an important biological variable that reflects disease aggressiveness in individual patients. The Oslo group demonstrated in their SECA-I study that a time interval of more than 2 years from primary tumor resection to LT was a significant favorable prognostic factor for OS, and this parameter was subsequently incorporated into the Oslo score [7]. Based on these findings, the subsequent SECA-II study required a minimum interval of 1 year from the diagnosis of colorectal cancer to LT as an eligibility criterion. Accordingly, the median intervals from diagnosis to LT and from primary tumor resection to LT were 24.0 and 22.6 months, respectively [8]. Similarly, the Toronto group adopted an interval of at least 6 months from primary tumor resection to LT as an eligibility criterion [28]. In this context, the International Hepato-Pancreato-Biliary Association consensus guidelines recommend a minimum of 1 year from the diagnosis of unresectable CRLM to LT [29]. Although the TransMet trial did not include this time interval as an eligibility criterion, the median interval between diagnosis and randomization for LT was 15.9 months. Furthermore, a prospective cohort study involving three North American liver transplant centers reported a median interval of 1.7 years from CRLM diagnosis to LT [30]. Collectively, these findings indicate that an interval of more than 1 year between diagnosis and LT is a widely accepted and clinically relevant eligibility criterion for LT for patients with unresectable CRLM.

### Response to chemotherapy

Response to chemotherapy is one of the most important prognostic factors for patients with colorectal cancer. For patients with advanced colorectal cancer who are not suitable for surgical treatment, the response rate to systemic chemotherapy is correlated with OS [31]. Moreover, it has been reported that patients with poor radiological or pathological responses to preoperative chemotherapy had shorter OS after hepatectomy for resectable CRLM [6, 32, 33]. For patients who have undergone LT, progressive disease during bridging chemotherapy is also a negative predictor of OS after surgery [19]. Therefore, the administration of systemic chemotherapy prior to LT is essential for assessing tumor biology and determining the suitability of patients for LT. In the SECA-II study, eligibility criteria included having received at least three cycles of chemotherapy (over 6 weeks) without tumor progression, regardless of the study arm. Notably, Arm C of the SECA-II study, which enrolled patients receiving second-line chemotherapy, required at least a 10% tumor response to second- or third-line chemotherapy [8]. Similarly, the TransMet trial required disease control for more than 3 months on the most recent chemotherapy regimen, which had to be third-line therapy or earlier [11]. Most other LT programs, including those in Japan, also require stable disease or a response to chemotherapy sustained over several months as a prerequisite for LT [10, 28, 34–36]. Consequently, the International Hepato-Pancreato-Biliary Association consensus guidelines recommend that a response to bridging therapy be observed for at least 6 months before LT and consider progressive disease during bridging therapy as a contraindication to LT [29]. In addition to assessing the response to chemotherapy as a prognostic marker, systemic chemotherapy in patients who are candidates for LT for unresectable CRLM might provide an opportunity to convert unresectable tumors to resectable tumors to avoid LT and to enable partial hepatectomy. In this context, achieving at least stable disease for more than 3 months during systemic chemotherapy represents a fundamental and widely accepted eligibility criterion for LT in patients with unresectable CRLM. Conversely, patients who exhibit radiologic disease progression should not be considered candidates for LT. At present, no specific chemotherapy regimens have been recommended universally for bridging therapy prior to LT across existing LT programs.

### Serum biomarkers and ctDNA

Changes in the levels of tumor markers, including carcinoembryonic antigen (CEA) and carbohydrate antigen (CA) 19-9, are prognostic markers in patients who undergo hepatectomy for CRLM. An increase in CEA or CA19-9 level during preoperative chemotherapy is reported to be associated with worse survival after hepatectomy [37, 38]. With respect to LT, the CEA level at the time of LT is associated with survival after transplantation [19]. Although no study has investigated the impact of changes in CEA levels during pretransplant chemotherapy on survival, the International Hepato-Pancreato-Biliary Association consensus guidelines recommend that high CEA levels (> 80 µg/L) with an increasing trend during chemotherapy should be considered as a strong relative contraindication for LT [29]. Ongoing clinical trials, including the SECA-III study (NCT03494946), the METLIVER study (NCT05398380), and the TRANSMETIR study (NCT04616495), explicitly include a CEA > 80 ng/mL at the time of enrollment as an exclusion criterion. The inclusion criteria of the Toronto group also included stable or decreasing CEA levels at all time points before transplantation [28]. Consequently, patients with increasing CEA levels during bridging therapy are generally considered poor candidates for LT. Given that no case series of patients who underwent LT for CRLM has evaluated the prognostic impact of CA19-9 levels or how these levels change during chemotherapy, the role of the perioperative CA19-9 level and its dynamics remain controversial and it should not apply when determining the indication for LT in current clinical practice.

Circulating tumor DNA (ctDNA) refers to cell-free DNA fragments in the bloodstream that originate from tumor cells through apoptosis, necrosis, or active secretion [39]. ctDNA has gained increasing attention as a form of liquid biopsy, with potential applications including the early detection of postoperative recurrence or metastases not identifiable by conventional surveillance methods, risk stratification for postoperative recurrence through detection of minimal residual disease to guide individualized adjuvant therapy, real-time assessment of tumor burden, and characterization of tumor-specific genomic alternations [39, 40]. Furthermore, ctDNA testing enables the real-time monitoring of various tumor-related parameters, particularly for colorectal cancer. Because ctDNA levels often change more rapidly than radiologic tumor volume during chemotherapy, ctDNA is expected to serve as a dynamic and sensitive biomarker for treatment response in this clinical setting [41]. Although next-generation sequencing (NGS)-based ctDNA assays are used widely in clinical practice for precision oncology, high-level evidence supporting the clinical utility of ctDNA testing to monitor response to chemotherapy in colorectal cancer remains limited. In relation to LT for CRLM, no prospective studies have evaluated the role of ctDNA in establishing transplant eligibility. A retrospective analysis from the Cleveland Clinic examined perioperative ctDNA dynamics in five patients with CRLM who underwent LT and found that 75% of patients with detectable preoperative ctDNA became ctDNA-negative following transplantation. These findings suggest that the presence of preoperative ctDNA should not necessarily preclude LT for patients with CRLM [42]. Currently, the International Hepato-Pancreato-Biliary Association consensus guidelines do not recommend the use of ctDNA for determining LT eligibility in patients with unresectable CRLM [29]. In this context, the role of ctDNA-based liquid biopsy in LT for CRLM remains investigational, and further prospective clinical studies are required before it can be applied in clinical practice.

### Gene mutation and microsatellite instability

Gene mutation profiles, such as those of RAS and BRAF, and microsatellite instability (MSI) or mismatch repair deficiency are associated with prognosis after hepatectomy for resectable CRLM [43–45]. Among patients with CRLM who have received systemic chemotherapy or undergone hepatectomy, 18–52% harbor an RAS mutation, and 1–5% harbor a BRAF mutation [43–46]. RAS is the most commonly recognized gene mutation relevant to the treatment of colorectal cancer and is associated with early lung recurrence and worse RFS and OS after hepatectomy [45, 47]. However, as RAS mutation alone does not preclude surgical intervention, selected patients with mutant RAS have also been considered for LT. BRAF mutation is strongly associated with the poor survival of patients with advanced colorectal cancer treated with palliative chemotherapy [48]. BRAF mutation is also reported to be associated with poor prognosis after hepatectomy, which is characterized by early death within 1 year after surgery [49]. In this context, the indication for hepatectomy in patients with BRAF-mutant CRLM remains a matter of debate [43, 44]. Given that patients with BRAF mutations are contraindicated for LT in the majority of LT programs for CRLM [8, 11], there is little evidence regarding posttransplant prognosis for unresectable CRLM harboring BRAF mutations [50]. Because of the shorter OS of BRAF V600-mutant patients than of non-V600 BRAF-mutant patients, the International Hepato-Pancreato-Biliary Association consensus guidelines recommend excluding patients with BRAF V600E-mutated CRLM from LT [29]. However, BRAF mutation, including the V600E mutation, was not considered an exclusion criterion in one of the largest prospective studies of LT for CRLM: the SECA-II study [8]. In contrast to these two genes, MSI-high status is known to be a favorable biomarker for treatment with immune checkpoint inhibitors (ICIs) for metastatic colorectal cancer, yielding a high response rate and longer OS [51]. Given the high response rate to ICIs, as well as the risk of graft rejection associated with ICI therapy after LT, the International Hepato-Pancreato-Biliary Association consensus guidelines recommend excluding these patients from LT for unresectable CRLM at the time of publication [29]. Conversely, another study reported that the presence of liver metastases is associated with poor response and survival even in MSI-high patients with metastatic colorectal cancer, who were treated with ICIs [52]. Therefore, this field remains underdeveloped, and further clinical trials are warranted to clarify and potentially redefine the indications for LT in MSI-H patients.

### Metabolic Tumor Volume from FDG-PET

Fluorine-18 fluorodeoxyglucose positron emission tomography with computed tomography (FDG-PET/CT) provides information not only about distant metastases, but also about functional/metabolic cancer activity in addition to anatomical information from CT or magnetic resonance imaging (MRI). FDG activity is usually quantified using the standardized uptake values (SUV) within a volume of interest (VOI), with higher SUVs reflecting increased tumor metabolic activity and aggressiveness. The Oslo group proposed the total metabolic tumor volume (MTV), defined as the tumor volume with 18 F-FDG uptake segmented using fixed threshold methods of 40% of SUVmax within the VOI, as well as total lesion glycolysis (TLG), calculated as SUVmean multiplied by MTV. These FDG-PET/CT-derived parameters have been reported to be strong prognostic markers for PFS, OS, and survival after relapse for patients who have undergone LT for unresectable CRLM, irrespective of the number of tumors [53, 54]. Specifically, a retrospective analysis of the combined SECA-I and SECA-II cohorts demonstrated that patients with an MTV < 70 cm^3^ had a significantly higher 5-year OS rate than those with an MTV ≥ 70 cm^3^ (78% vs. 22%, *p* = 0.001) [18]. Similarly, an analysis of the combined cohorts of SECA-I, SECA-II (arms A, B, C and D), and the RAPID study revealed a significantly higher 5-year OS rate for patients with an MTV < 70 cm^3^ than for those with an MTV ≥ 70 cm^3^ (66.7% vs. 23.3%, *p* < 0.001) [19]. Moreover, a pre-transplant TLG > 260 g was significantly associated with shorter OS and DFS [54]. The fact that patients enrolled in SECA-II had lower median MTV and TLG values than those in SECA-I study reflects more stringent patient selection in the SECA-II study (21.3 vs. 98.5 cm^3^, *p* = 0.08; 76 vs. 98.5 g, *p* = 0.06, respectively) [8]. Furthermore, a multicenter study from the US externally validated the predictive efficacy of the MTV assessed by FDG-PET/CT and proposed it as a key selection criterion for LT in patients with CRLM [55]. These results indicate that FDG-PET/CT-based assessment of systemic therapy response reflects biological activity as well as a size-based response assessment, according to response evaluation criteria for solid tumors (RECIST) [56]. Consequently, International Hepato-Pancreato-Biliary Association consensus guidelines recommend excluding patients with an MTV > 70 cm^3^ and a TLG > 260 g from LT for CRLM [29]. It should be noted that the most recent clinical trial conducted by Oslo group (SECA-III; NCT03494946) does not include metabolic tumor burden assessed by FDG-PET as an inclusion criterion. Similarly, the TransMet trial and the UKCoMET study do not apply metabolic tumor burden assessed by PET/CT as an eligibility criterion, but instead record it as a variable of interest for registry-based analysis [11, 34].

### Perihepatic lymph node metastasis

Perihepatic lymph node metastasis (PHLM), including lymph node metastases along the hepatoduodenal ligament (Station 12), common hepatic artery (Station 8), coeliac trunk (Station 9) and in the aortocaval space (Station 16), is considered to represent metastatic spread originating not from the primary tumor but from the CRLM themselves, and is one of the poorest prognostic factors for patients with CRLM. The 5-year OS rate after hepatectomy for patients with a combination of PHLM and CRLM was reported to be less than 10%; thus, it was considered a contraindication for hepatectomy in the 1990s [57, 58]. Conversely, in the era of modern chemotherapy and molecular targeted agents, encouraging outcomes have been reported, with 5-year OS rates ranging from 13 to 21% after hepatectomy. Although there have been no randomized trials comparing hepatectomy and perihepatic lymphadenectomy with chemotherapy alone, the results of recent studies support the benefit of lymphadenectomy for selected patients [59–62]. However, it is uncertain whether the results of recent studies of patients with resectable CRLM and PHLM can be applied to patients with unresectable CRLM who are candidates for LT.

**Fig. 1:**
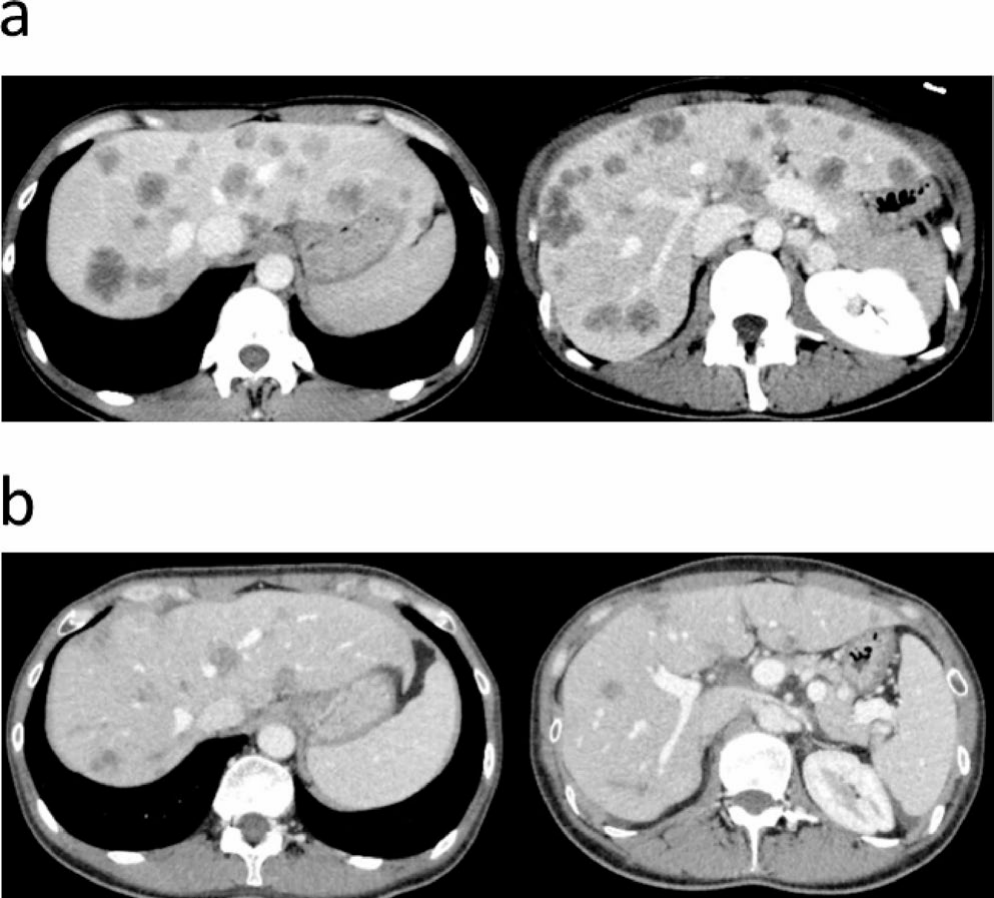
Computed tomography (CT) images before (**a**) and after (**b**) systemic chemotherapy of a patient who underwent liver transplantation for unresectable colorectal liver metastases. The size of the tumors decreased remarkably after chemotherapy, but remained unresectable

The reported incidence of PHLM in patients with resectable CRLM varies depending on preoperative radiological assessment and macroscopic assessment during surgery. The incidence of pathologically confirmed PHLM was 40–50% in patients with radiologically suspected PHLM and 20% in patients with intraoperatively suspected PHLM despite the absence of signs of PHLM on preoperative imaging [62, 63]. PHLM is still confirmed pathologically in 5%−18% of patients without signs on preoperative imaging or macroscopic findings during surgery [62, 64–66]. The MD Anderson Cancer Center group reported that the OS of patients without PHLM confirmed by lymphadenectomy was significantly better than that of patients who did not undergo lymphadenectomy [62]. Therefore, PHLM should also be suspected in patients who are candidates for LT for unresectable CRLM even if there is no suspicion of PHLM. Risk factors for PHLM include primary tumor LN metastases, synchronous liver metastases, intrahepatic recurrence requiring repeat hepatectomy, three or more liver metastases, nodules located in segments 4 and 5, elevated CEA levels, and peritoneal metastases [62, 67, 68]. Given the high tumor burden in candidates for LT for CRLM, which includes a high number of tumors, tumors located in segments 4 and 5, and elevated CEA levels, these patients may have a higher likelihood of PHLM than those with resectable CRLM (Fig. 1).

**Fig. 2:**
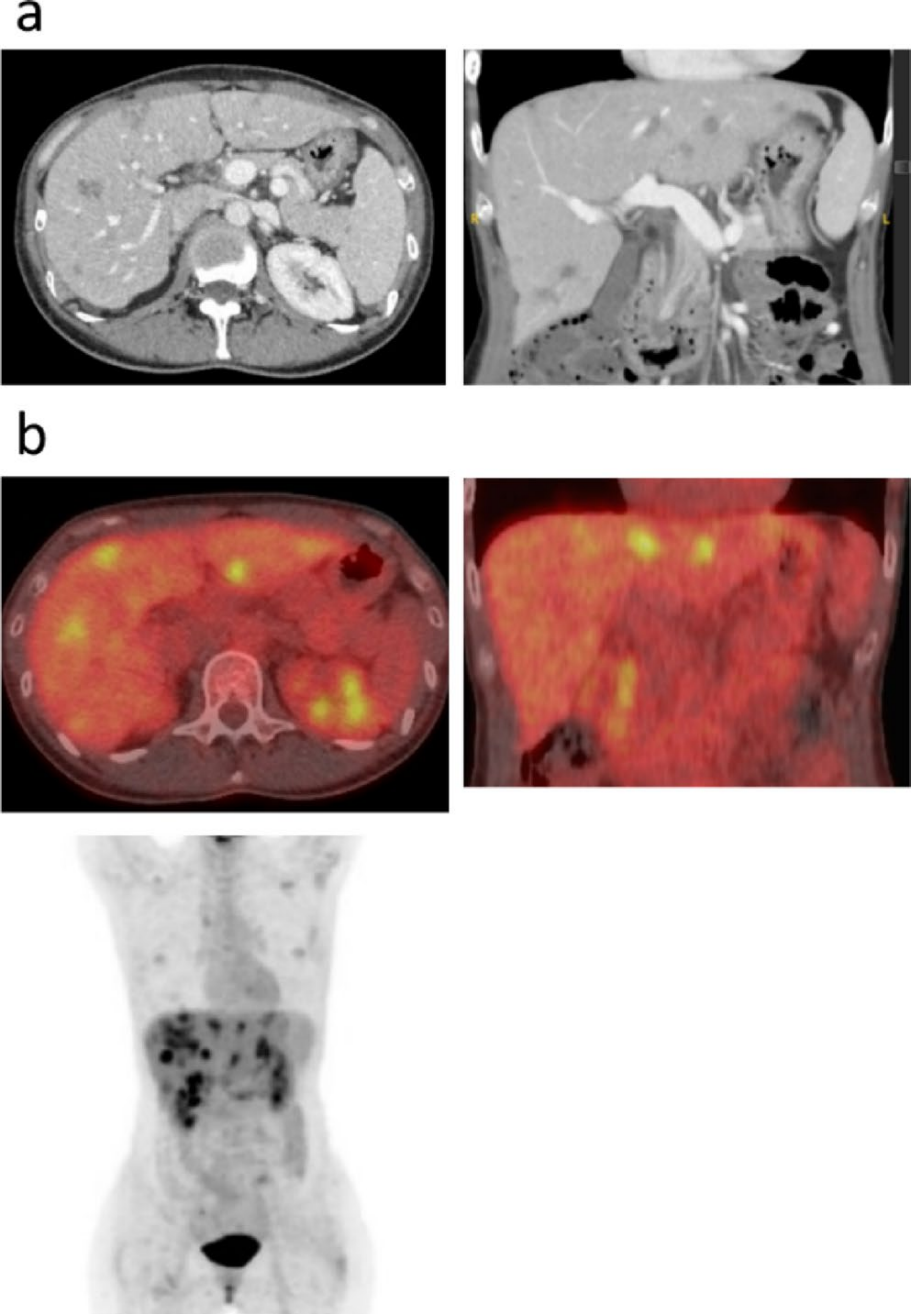
Computed tomography (CT) and fluorine-18 fluorodeoxyglucose positron emission tomography (FDG-PET) images before liver transplantation. Although this patient had pathological positive lymph node metastasis at Station 12b, no perihepatic lymph node swelling was identified on CT or PET

Because of the higher incidence of PHLM related to increased tumor burden and the higher postoperative recurrence rate, the indication for LT in patients with PHLM should be determined with caution. The International Hepato-Pancreato-Biliary Association consensus guidelines for LT for nonresectable colorectal liver metastases recommend that patients with lymph node-positive disease be excluded from LT [29]. If the PHLM is considered extrahepatic, these patients are also contraindicated for LT according to the criteria of the Japanese multi-institutional clinical trial on living-donor LT for CRLM [10]. Another clinical issue is whether to perform routine intraoperative nodal sampling before LT. Given the low positive predictive value (30–56%) and sensitivity (33–40%) of CT for detecting PHLM [62, 69, 70], the results of preoperative CT assessments are considered inconclusive (Fig. 2). Although the positive predictive value of FDG-PET is reported to be 100%, the number of patients with PET-positive perihepatic lymph nodes in these studies was small [69]. Therefore, nodal sampling should be considered, at least for patients with suspected PHLM, based on preoperative imaging and/or on intraoperative findings. Given that patients who are candidates for LT often have a higher likelihood of PHLM than those with resectable CRLM, a frozen-section examination of perihepatic lymph nodes should be considered before initiating donor surgery, although supporting evidence is limited. In the SECA-I study, the Oslo group routinely performed a staging laparotomy with examination of frozen sections of lymph nodes in the hepatic ligament and adjacent tissue, even when there was no sign of metastases on preoperative CT scans [7].

### Limitations

Several limitations of the current review should be acknowledged. First, most of the included studies that formed the basis for prognostic factors and selection criteria were limited by a small sample size. Second, most of the data referenced in this review were derived from single-center, institution-specific experiences, particularly from the Oslo group. This predominance may introduce selection bias, and the findings of prior studies require external validation. Detailed analyses from the TransMet trial are highly anticipated. Furthermore, results from ongoing LT programs in Japan, North America, Europe, and East Asia may help validate the proposed prognostic factors and scoring systems and potentially identify novel prognostic markers.

## Conclusion

Several prognostic factors are used for selecting good candidates for LT for CRLM. These include tumor burden, extrahepatic metastases, gene mutation profile, and response to chemotherapy. This review also discusses prognostic factors that are not currently incorporated into standard LT program criteria but may serve as novel eligibility markers to improve patient selection, including metabolic tumor volume assessed by PET/CT, ctDNA, and the presence of perihepatic lymph node metastases (Table 2). Considering the scarcity of donor organs for deceased-donor LT, the need to ensure the safety of living donors, and the goal of achieving better outcomes, appropriate candidates for LT for unresectable CRLM should be selected on the basis of these factors. Conversely, the ongoing SECA-III study has adopted expanded eligibility criteria regarding extrahepatic disease, allowing patients with resectable pulmonary metastases < 15 mm to be considered for LT. This approach is based on findings from the SECA-II study, which demonstrated relatively favorable post-recurrence outcomes when recurrence was limited to the lungs [8]. Further clinical studies and results from ongoing LT trials are needed to validate the clinical utility of these extended eligibility criteria.

**Table 2:** Factors for optimal patient selection to achieve a good prognosis after liver transplantation for unresectable colorectal liver metastases

Scoring systems	Fong score 0-1
	Oslo score 0
Biological factors	Left-sided primary tumors
	Interval between diagnosis and liver transplantation > 1 year
Response to chemotherapy	Radiological response or stable disease during chemotherapy for 6 months before liver transplantation
Serum biomarkers	Stable or decreased trend in CEA level during chemotherapy
Gene mutation and MSI	BRAF-V600 wild-type
	MSI-low or MSS*
MTV from FDG-PET	MTV <70 cm3
	TLG >260 g
Perihepatic lymph node metastasis	No perihepatic lymph node metastasis

CEA, carcinoembryonic antigen; MSI, microsatellite instability; MSS, microsatellite stable; MTV, metabolic tumor volume; FDG-PET, fluorine-18 fluorodeoxyglucose positron emission tomography; TLG, total lesion glycolysis
